# Drought Tolerance Responses in Vegetable-Type Soybean Involve a Network of Biochemical Mechanisms at Flowering and Pod-Filling Stages

**DOI:** 10.3390/plants10081502

**Published:** 2021-07-22

**Authors:** Makoena Joyce Moloi, Rouxlene van der Merwe

**Affiliations:** 1 Department of Plant Sciences-Botany Division, University of the Free State, 205 Nelson Mandela Drive, Park West, Bloemfontein 9301, South Africa; 2 Department of Plant Sciences-Plant Breeding Division, University of the Free State, 205 Nelson Mandela Drive, Park West, Bloemfontein 9301, South Africa; vandermerwer@ufs.ac.za

**Keywords:** antioxidative enzymes, edamame, *Glycine max*, hydrogen peroxide, lipid peroxidation, proline, total soluble sugars, water deficit

## Abstract

Severe drought stress affects the production of vegetable-type soybean (*Glycine max* L. Merrill), which is in infancy for Africa despite its huge nutritional benefits. This study was conducted under controlled environmental conditions to establish the effects of severe drought stress on ascorbate peroxidase (APX), guaiacol peroxidase (GPX), and glutathione reductase (GR) activities as well as proline, total soluble sugars (TSS), and hydrogen peroxide (H_2_O_2_) contents of five vegetable-type soybean cultivars (UVE8, UVE14, UVE17, AGS354, AGS429) at flowering and pod-filling stages. Drought induced significant increases in the contents of proline (selectively at pod filling for AGS429), TSS (at both stages for AGS429, and only at pod filling for UVE14), and malondialdehyde (AGS354 at flowering; UVE17 at pod filling). UVE8 and AGS354 had the highest H_2_O_2_ levels at flowering under drought stress, while AGS429 had the lowest. However, AGS429 was the only cultivar with significantly increased H_2_O_2_ under drought stress. Furthermore, drought stress induced significant increases in APX, GPX, and GR activities at flowering for AGS429. AGS354 recorded the highest decline for all antioxidative enzymes, while UVE17 decreased for GPX only. All biochemical parameters, except H_2_O_2_, were significantly higher at pod filling than at the flowering stage. The relationship between H_2_O_2_ and total seed mass (TSMP) or total seed per plant (TSP) was significantly positive for both stages, while that of TSS (at flowering) and proline (at pod filling) were significantly related to total pods per plant (TPP). The study suggests that during drought, the tolerance responses of vegetable-type soybean, APX, GPX, and GR (especially at the flowering stage), function in concert to minimize H_2_O_2_ production and lipid peroxidation, thereby allowing H_2_O_2_ to function in the signaling events leading to the induction of drought tolerance. The induction of TSS at flowering and proline at pod filling is important in the drought tolerance response of this crop.

## 1. Introduction

Drought stress is a catalyst of great famines and is accelerated by anthropogenic climate changes [1]. Such stress negatively affects soybean production [2]. Vegetable-type soybean (*Glycine max* L. Merrill) is a complete protein food source (because it contains all eight essential amino acids) with numerous vitamins, minerals, isoflavones, fiber, magnesium, folate; it also lowers blood cholesterol [3]. Production of this crop in Africa is in its infancy despite its huge nutritional advantages. Only a few cultivars are produced by small-scale, resource-poor farmers [4]; these cultivars are not yet commercialized in South Africa. Since the crop is less popular, knowledge about its responses to extreme environmental conditions such as drought is inadequate. This deepens the threat of food security in Africa and other parts of the world [5]. 

The responses of plants to drought stress varies from species to species, depending on plant growth stage and other environmental factors [6]. Mild stress at the grain filling phase can substantially reduce crop yield. Other noticeable effects of drought stress include damage of the photosynthetic apparatus, membrane instability [7], and, consequently, death, which mainly result from oxidative stress caused by excessive production of reactive oxygen species (ROS) such as superoxide radicals and hydrogen peroxide [8]. Plants have evolved a number of biochemical and physiological mechanisms to improve their tolerance to abiotic stress. These may include the employment of various antioxidative mechanisms that are enzymatic or non-enzymatic in nature. Some antioxidative mechanisms include enzymes of the ascorbate–glutathione pathway, guaiacol peroxidase, catalase, and superoxide dismutase [9]. In addition, plants may also cope with drought stress by stimulating the production of osmolytes such as proline and non-structured sugars, which increase their osmotic potential [1]. 

Knowledge of the biochemical mechanisms for drought tolerance in vegetable-type soybeans is very limited. In order to improve the tolerance of this crop to drought, which is the ultimate goal, a better understanding of the physiological and biochemical mechanisms of drought tolerance is key because such information may be used for the selection of better-performing cultivars. Previous studies on grain soybean have suggested that yield can be associated with high proline accumulation under water limiting conditions [10]. Furthermore, a susceptible grain soybean cultivar with low proline content has corresponding low ascorbate peroxidase activity at both flowering and pod-filling stages [11]. However, no information is available on the biochemical responses of vegetable-type soybean cultivars used in this study. Therefore, this research aims to establish the important biochemical mechanisms of drought tolerance in vegetable-type soybean. The study was performed at flowering and pod-filling stages to determine if such biochemical responses would differ according to the developmental stages under drought stress in this crop. Additionally, the study provides information on the biochemical performance of the various cultivars under drought stress. Such information will accelerate vegetable-type soybean drought tolerance breeding, which will improve crop yield (tons per hectare) under severe drought stress. 

## 2. Results

At the flowering stage, the cultivar effect was significant for all parameters except for proline. The water treatment effect was not significant for GPX, GR, and proline. Cultivar by treatment interaction was significant for the antioxidative enzymes and TSS. At pod filling, the cultivar effect was not significant for TSS, while the treatment effect was not significant for GR. Cultivar by treatment effect was significant for the antioxidative enzymes only (Table 1).

**Figure 1:**
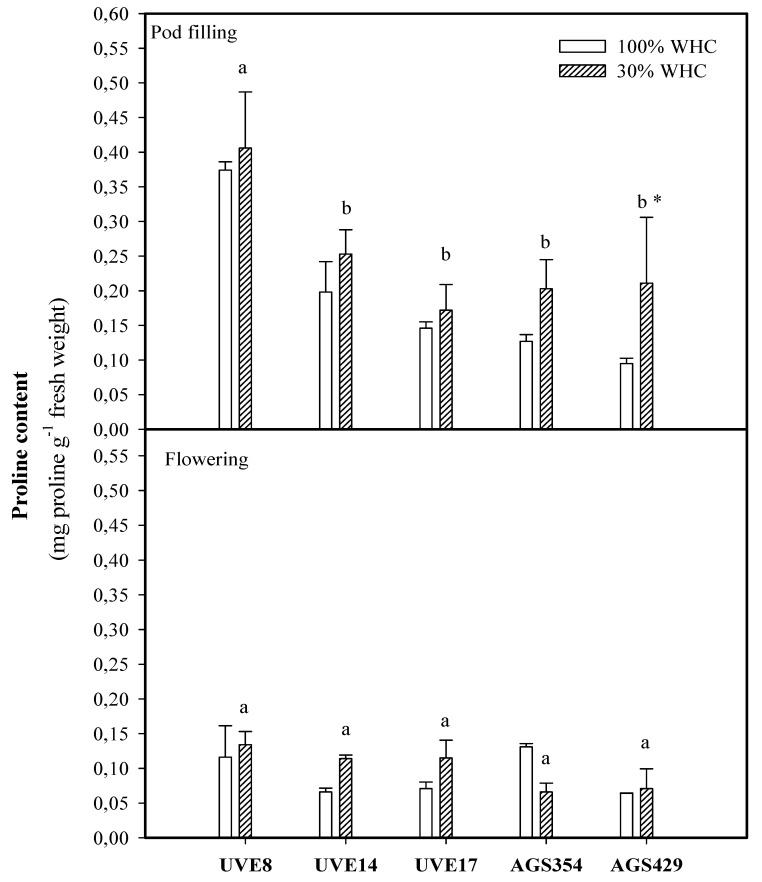
Proline content for five vegetable-type soybean cultivars subjected to two water treatments at flowering and pod filling stages. Values represent means ± SD (*n* = 3). Letters represent significant differences/similarities in proline content for various cultivars under severe water stress at 30% water holding capacity (WHC), * represents significance at *p* ≤ 0.05.

At flowering, drought stress increased proline accumulation to higher levels in all cultivars with the exception of AGS354. However, such responses were not significant. Moreover, there was no distinction between the cultivars under drought stress treatment at the flowering stage. Although cultivars were not significantly different under drought stress (except for UVE8) at pod filling, proline increased to substantiated levels in AGS429 (2.25-fold). Proline was lower at flowering than at pod filling (Figure 1). 

**Figure 2:**
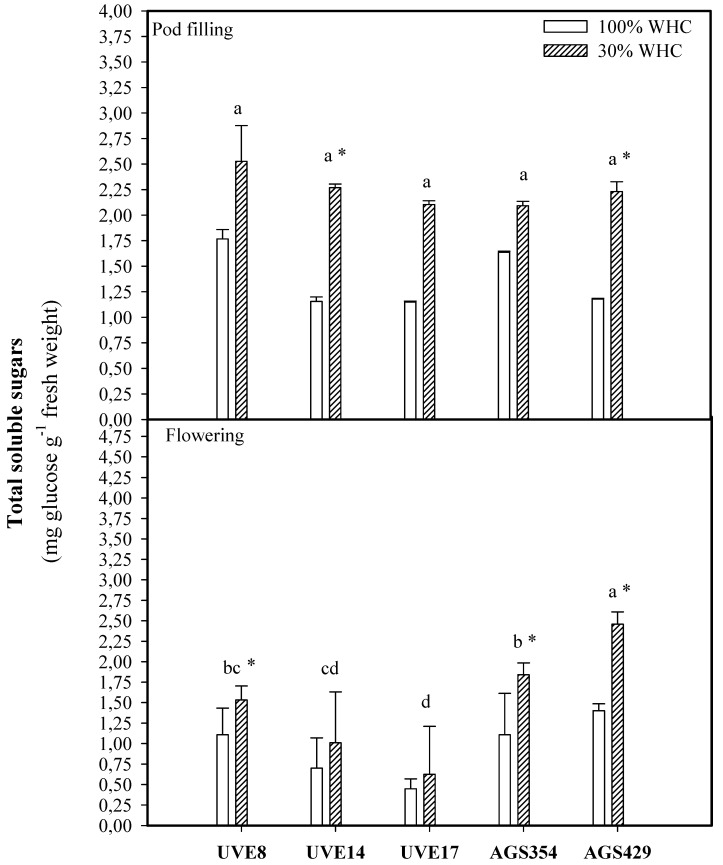
Total soluble sugar (TSS) content for five vegetable-type soybean cultivars subjected to two water treatments at flowering and pod-filling stages. Values represent means ± SD (*n* = 3). Letters represent significant differences/similarities in TSS content for various cultivars under severe water stress at 30% water holding capacity (WHC), * represents significance at *p* ≤ 0.05.

Drought stress induced TSS production at flowering, with the highest significant increase observed for AGS429 (1.75-fold). This cultivar was significantly different from others under drought stress at this stage. At pod filling, under drought stress, the cultivars were not significantly different. However, the increase in TSS production due to drought stress was significantly high in AGS429 (47% increase) and UVE14 (49% increase) compared to the controls. Higher TSS values were recorded at flowering compared to the pod-filling stage (Figure 2).

**Figure 3:**
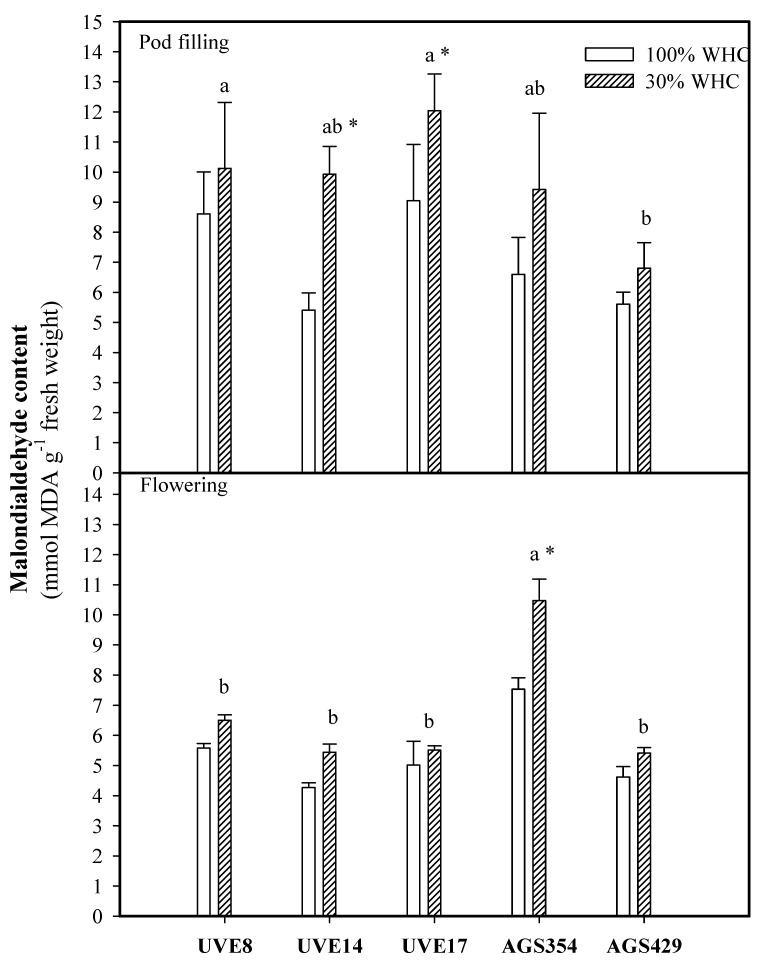
Malondialdehyde (MDA) content for five vegetable-type soybean cultivars subjected to two water treatments at flowering and pod-filling stages. Values represent means ± SD (*n* = 3). Letters represent significant differences/similarities in MDA content for various cultivars under severe water stress at 30% water holding capacity (WHC), * represents significance at *p* ≤ 0.05.

Malondialdehyde (MDA) content was not significantly different for all cultivars except AGS354 under drought stress at flowering. The highest and most significant increase in MDA production was observed in AGS354 (1.39-fold). At pod filling, a similar pattern was observed, where drought stress induced the production of MDA in all cultivars. However, UVE17 and UVE14 were the only two cultivars with significant increases in MDA (1.37-fold and 1.84-fold, respectively) compared to the control (Figure 3). 

**Figure 4:**
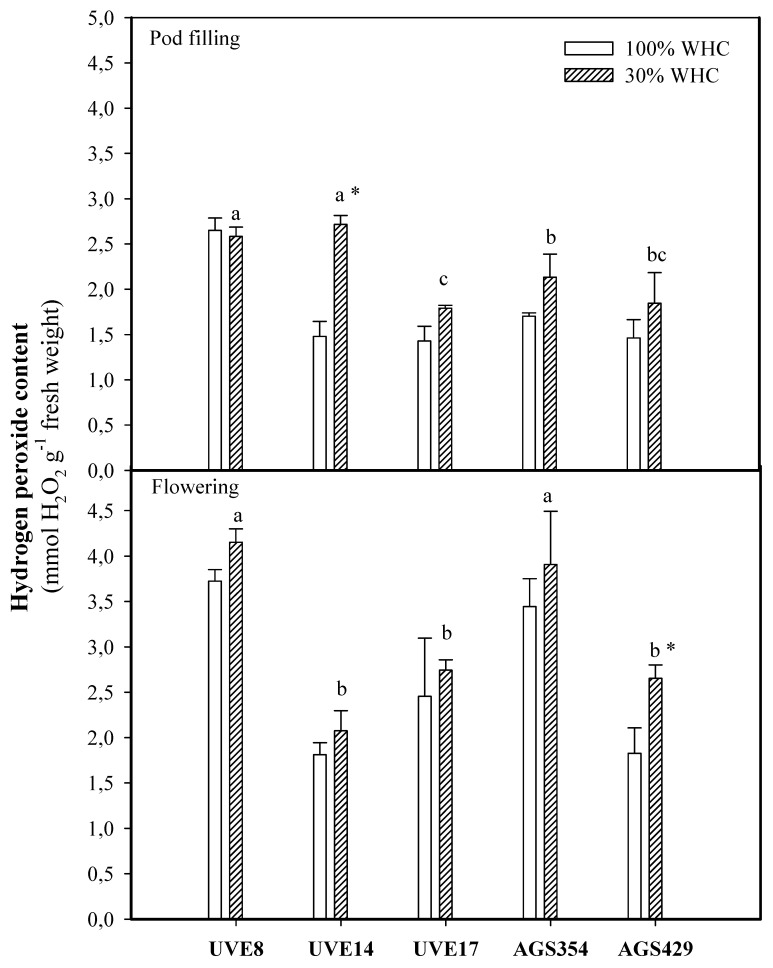
Hydrogen peroxide contents for five vegetable-type soybean cultivars subjected to two water treatments at flowering and pod-filling stages. Values represent means ± SD (*n* = 3). Letters represent significant differences/similarities in H_2_O_2_ content for various cultivars under severe water stress at 30% water holding capacity (WHC), * represents significance at *p* ≤ 0.05.

Hydrogen peroxide content increased with drought stress irrespective of the growth stage except for UVE8 at the pod-filling stage. Although the level of H_2_O_2_ was low in AGS429, it was the only cultivar with significantly increased H_2_O_2_ (1.45-fold) compared to the control. Under drought stress at pod filling, UVE14 was the only cultivar that induced a substantial increase in H_2_O_2_ content (1.62-fold) compared to the control (Figure 4). 

**Figure 5:**
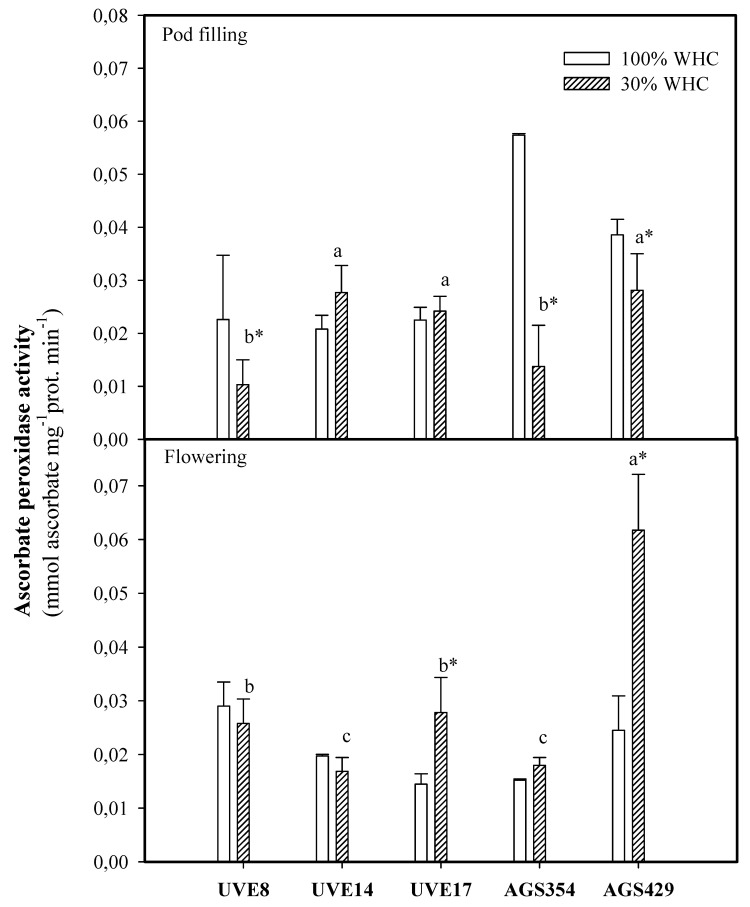
Ascorbate peroxidase (APX) activity for five vegetable-type soybean cultivars subjected to two water treatments at flowering and pod-filling stages. Values represent means ± SD (*n* = 3). Letters represent significant differences/similarities in APX activity for various cultivars under severe water stress at 30% water holding capacity (WHC), * represents significance at *p* ≤ 0.05.

Drought stress induced a substantial increase in the APX activity of UVE17 (47%) and AGS429 (60%) at flowering. AGS429 was significantly high and different from the other cultivars for this parameter under drought stress at flowering. At pod filling, there was a significant decrease in the APX activity of UVE8, AGS354, and AGS429 under drought stress, with the highest decrease recorded for AGS354 (76%). Although not significant, there was a slight increase in the APX activity of UVE14 under drought stress (Figure 5).

**Figure 6:**
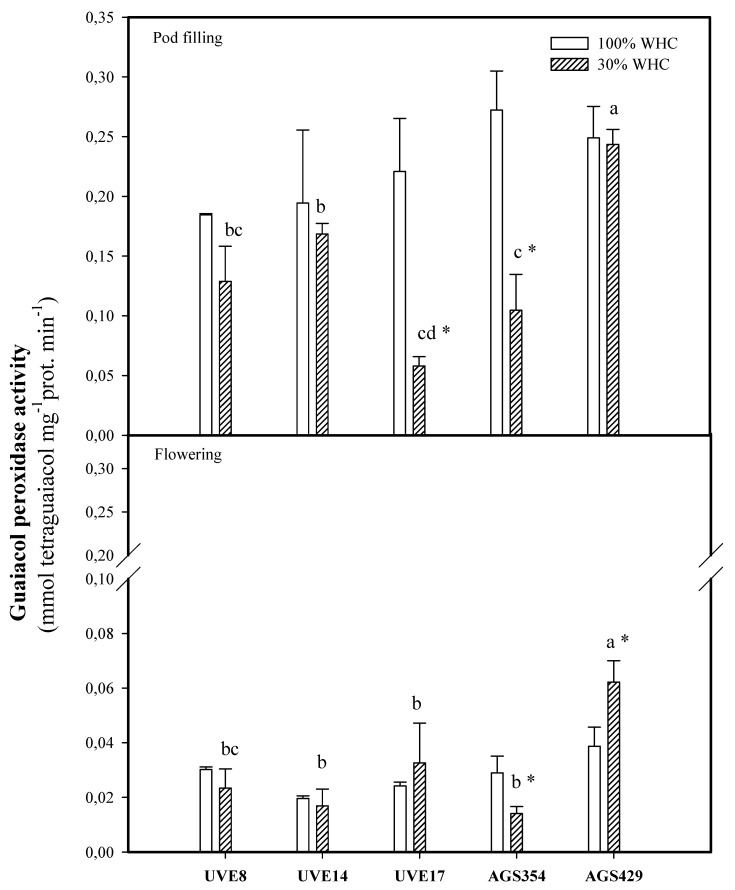
Guaiacol peroxidase (GPX) activity for five vegetable-type soybean cultivars subjected to two water treatments at flowering and pod-filling stages. Values represents means ± SD (*n* = 3). Letters represent significant differences/similarities in GPX activity for various cultivars under severe water stress at 30% water holding capacity (WHC), * represents significance at *p* ≤ 0.05.

There was a significant drop in GPX activity of drought-stressed AGS354 (66%) at flowering. Contrarily, AGS429 had a substantial increase (45%) in GPX activity under drought stress. At pod filling, there was a decline in GPX activity under drought stress, where UVE17 and AGS354 recorded significant decreases (74% and 62%). Interestingly for AGS429, there was no decrease under drought stress. AGS429 was different from the other cultivars at flowering and pod filling under drought stress (Figure 6).

**Figure 7:**
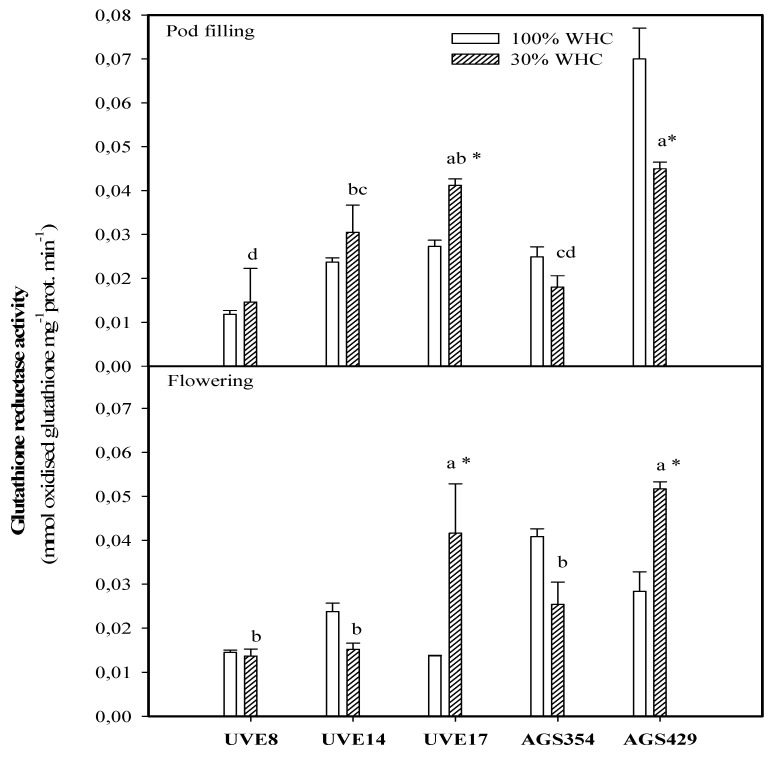
Glutathione reductase (GR) activity for five vegetable-type soybean cultivars subjected to two water treatments at flowering and pod-filling stages. Letters represent significant differences/similarities in GR activity for various cultivars under severe water stress at 30% water holding capacity (WHC), * represents significance at *p* ≤ 0.05.

AGS429 and UVE17 were not different for GR activity at both flowering and pod-filling stages under drought stress. At flowering, drought stress induced substantial increases in the GR activity of UVE17 (70%) and AGS429 (45%). At pod filling, a significant increase in GR activity was only for drought-stressed UVE17 (33%). In contrast to the flowering stage, drought stress substantially reduced the GR activity of AGS429 by 34% (Figure 7).

Correlations between the yield parameters and biochemical responses were analyzed to determine their relationships under drought stress conditions. At flowering, there were highly significant positive correlations (*p* ≤ 0.01) between H_2_O_2_ and TSMP as well as TSP. Additionally, TSS correlated positively with TPP (*p* ≤ 0.05). At pod filling, there was a significant but negative correlation between APX and TSMP and TSP (*p* ≤ 0.05). Likewise, GR was negatively correlated to these parameters in a significant manner. There was a noteworthy positive correlation between H_2_O_2_ and TSP (*p* ≤ 0.05). Proline was significantly correlated with TSP (Table 2).

Out of the 11 principal components (PCs), PC1 and PC2 explained 76.12% of the total variation for cultivars under severe drought stress during flowering. Parameters that showed positive loadings with PC1 were H_2_O_2_, MDA, TPP, TSMP, TSS, and TSP. The positive associations among the parameters were in accordance with results from Table 2, where significant positive correlations were observed between H_2_O_2_ with TSMP and TSP, respectively, and between TSS and TPP. At pod filling, PC1 and PC2 explained 73.09% of the total variation under severe stress. Parameters that showed positive loadings with PC1 were H_2_O_2_, proline, TSMP, TSS, and TSP. From these parameters, only TSP showed significant positive correlations with H_2_O_2_ and proline, respectively (Table 2). However, TSMP and TSP, which showed positive loadings in PC1, were significantly and negatively correlated with APX and GR, which showed negative loadings in PC1.

**Figure 8:**
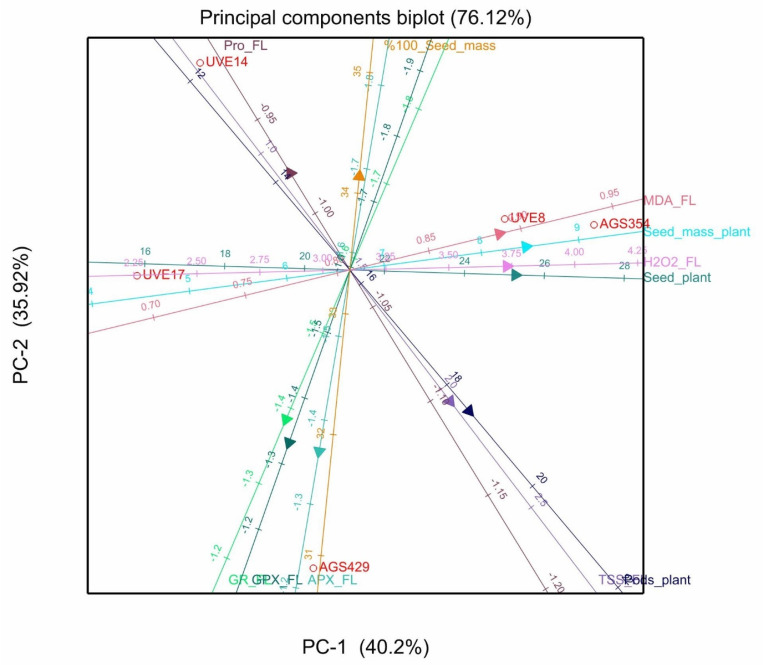
Principal components biplot between PC1 and PC2 showing the contribution of biochemical and yield parameters to the variability of five vegetable-type soybean cultivars subjected to severe stress at flowering.

Results from the PCA biplots confirmed the variability among cultivars in response to the biochemical parameters under severe drought stress. At flowering, cultivar AGS429 showed the highest production for TSS, APX, GPX, and GR, while cultivar AGS354 had the highest significant contents of MDA and H_2_O_2_ (Figure 8). 

**Figure 9:**
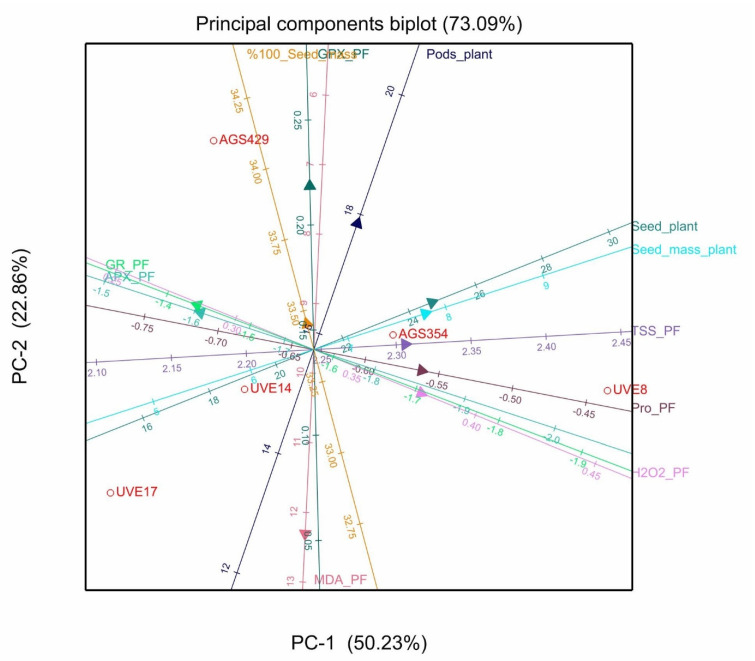
Principal components biplot between PC1 and PC2 showing the contribution of biochemical and yield parameters to the variability of five vegetable-type soybean cultivars subjected to severe stress at pod filling.

At pod filling, cultivar UVE8 showed the highest production of proline, TSS, and H_2_O_2_, while it showed lower production of APX and GPX. Cultivar AGS429 had the highest production of GPX and GR, with the lowest production of MDA content (Figure 9).

## 3. Discussion

Changing climatic conditions, such as increases in drought occurrences globally, are detrimental to crop growth and production because they hinder plant metabolic functions [12]. Proline often plays an important osmoregulation role, especially during drought stress [1]. Moreover, it can also act as an antioxidant in plants, thereby preventing oxidative stress and stabilizing cell membranes [13]. In peanuts, drought tolerance was associated with a high accumulation of proline biosynthesis [14]. Similarly, increased proline accumulation corresponded to less reduction in the relative water content and shoot and root fresh/dry weight in Indian grain soybean cultivars exposed to mild water stress [15]. Contrarily, our study shows that drought stress does not affect proline accumulation for all cultivars at flowering. It was only at pod filling that drought stress induced a significant increase in proline accumulation, which was selective for AGS429 (Figure 1). This agrees with a review by Mwenye et al. [16], who showed that genotypic variations in proline accumulation exist in soybeans. In addition, higher proline accumulation at pod filling shows that its induction under drought stress is specific for AGS429 at this growth stage, which is in agreement with findings that a plant’s response to drought stress varies with species and developmental stage [6]. Based on the significant positive correlation between proline at pod filling and total seeds per plant (TSP) (Table 2), it is evident that upregulation of proline accumulation at this stage could be crucial for some of the drought tolerance responses of vegetable-type soybean. AGS429 is a stable cultivar with low yield reduction under drought stress [17]. Therefore, induction of proline during drought stress suggests its importance in the drought tolerance of vegetable-type soybean.

The non-structured sugars formed during photosynthesis are the primary sources of translocated and partitioned carbon in plants. Since drought stress affects the photosynthesis process negatively, it is common for plants to adjust their accumulation of soluble sugars (Nishizawa et al. 2008, as cited by [18]). The current study shows that cultivars reacted differently when it comes to total soluble sugars (TSS). Drought stress induced a high accumulation of TSS in AGS429 at flowering and pod filling, while it was significantly high only at pod filling for UVE14 (a stable cultivar under severe drought stress) (Figure 2). These findings suggest the involvement of TSS in the drought tolerance responses of vegetable-type soybean, probably in osmoregulation, stabilizing biomolecules and cell membranes [19] and preventing oxidative damage (Rolland et al. 2006, as cited by [18,20]). A significant relationship between TSS and total pods per plant (TPP) at flowering (*p* ≤ 0.05; Table 2, Figure 8) and a further strong association between these parameters at pod filling (Figure 9) show that non-structured sugars are highly significant for the induction of the drought tolerance responses in vegetable-type soybeans. Similar to proline, it appears that TSS content increased with the reproductive stages.

Like many environmental stresses, drought stress induces the overproduction of reactive oxygen species (ROS), leading to oxidative stress. This condition is harmful to plants as it can cause damage to biomolecules such as the lipids to the fatty acids of a cell membrane, producing small hydrocarbons such as malondialdehyde (MDA), the final product of lipid peroxidation and a sign of cellular membrane damage [21]. Drought stress induced significantly high MDA accumulation at flowering in AGS354. This suggests that severe drought stress induced oxidative stress in this cultivar, leading to high lipid peroxidation and poor yield performance. A study by van der Merwe et al. [17] supports this suggestion because they showed that AGS354 is a cultivar with a high yield reduction under severe drought stress. A high MDA content in AGS354 further supports this suggestion (Figure 8). Contrarily, the lower MDA content, with an insignificant increase under severe drought stress, in AGS429 (Figure 3) suggests that it has a better mechanism of scavenging ROS, thereby limiting oxidative-stress-related consequences. 

High accumulation of H_2_O_2_ in drought-stressed vegetable-type soybean cultivars (Figure 4) confirms that this type of stress induces an accumulation of ROS, which leads to oxidative stress and, consequently, lipid peroxidation. However, a relationship between H_2_O_2_ production and lipid peroxidation under drought stress needs consideration because the responses differ with the developmental stages. For example, at flowering under drought stress, AGS429 had the lowest MDA content, with a significant increase in H_2_O_2_ content (although the level of H_2_O_2_ was lower than that of other cultivars). Contrarily, at pod filling for UVE14, a significant increase in H_2_O_2_ production corresponded to a significant increase in lipid peroxidation. However, the correlation between these two parameters was significant at flowering but not at pod filling (Table 2, Figure 8). Since H_2_O_2_ is positively correlated with total seed mass per plant (TSMP) (Table 2) and total seed per plant (TSP) under drought stress (also confirmed by Figure 8 and Figure 9), which are the essential yield parameters, the results suggest that H_2_O_2_ is a very important biochemical parameter that is involved in the signaling events of vegetable-type soybean drought tolerance responses. However, the production of H_2_O_2_ needs strict control measures to avoid oxidative burst. This agrees with a study by Guler and Pehlivan [22], who reported that exogenous application of H_2_O_2_ at low doses elevates the drought tolerance responses of soybeans. Furthermore, exogenous H_2_O_2_ effectively alleviated oxidative damage in rice seedlings by upregulating the activities of antioxidant enzymes [23]. H_2_O_2_ is a key player in signal transduction pathways associated with abiotic stress when not produced excessively during abiotic stress [24]. Therefore, a threshold for the role of H_2_O_2_ as a signaling molecule in vegetable-type soybean needs to be established.

In order to maintain ROS balance under stress (i.e., to prevent oxidative stress), plants must possess a functional antioxidative system. The above findings on the involvement of H_2_O_2_ in the drought tolerance responses of vegetable-type soybean will prompt an understanding of the role of antioxidative enzymes in this regard. Studies of more than a decade ago revealed a positive correlation between drought tolerance and the ROS scavenging capacity of plants [25]. The level of increase or inhibition of antioxidative enzyme activities under drought stress is variable among plant species and even between cultivars of the same species [26]. In grain, soybeans under drought stress have induced ascorbate peroxidase (APX) activity, which serves as a biochemical marker for drought tolerance [11]. In the current study, AGS429 recorded the most significant increase in APX activity at the flowering stage. It appears that the effect varies with cultivars and developmental stages because, at pod filling, APX activity dropped for almost all cultivars under drought stress but not significantly for UVE14 (Figure 5). There was no direct link between APX activity at flowering and the drought tolerance responses (explained by insignificant correlations between APX and the yield parameters; Table 2 and Figure 8). 

At pod filling, this relationship was negative (Table 2, Figure 9). This indicates that higher APX activity is required to maintain homeostasis between H_2_O_2_ produced and scavenged, thereby allowing H_2_O_2_ to be involved in the signaling events for induction of drought tolerance responses in vegetable-type soybean. In agreement with this view, the highest decrease in APX activity for AGS354 (which is a highly unstable cultivar) at pod filling associates APX with vegetable-type soybean drought tolerance indirectly. An increase in the guaiacol peroxidase (GPX) activity of AGS429 was selective only for the flowering stage under drought stress. For other cultivars, GPX activity either decreased or was not significant (Figure 6). Supporting this, AGS354 and UVE17, which are susceptible to severe drought, had a huge reduction in GPX activity. Similar to APX, there was no significant difference in the GPX activity of AGS429 at pod filling, which indicates that for this cultivar, GPX is more effective at the flowering stage. This enzyme was not directly associated with any of the yield parameters. Under drought conditions, GR favors the maintenance of the glutathione pool, thereby intensifying the antioxidative response of the plant [27]. Although the glutathione reductase (GR) activity of AGS429 and UVE17 increased due to drought stress (Figure 7), significant but negative relationships between this enzyme and the yield parameters (TSMP and TSP) under drought stress (Table 2, Figure 8 and Figure 9) indicate that it is not directly responsible for the drought tolerance responses of vegetable-type soybean. Rather, along with the other antioxidative enzymes studied, they prevent lipid peroxidation and control H_2_O_2_ production to the level that can stimulate the signaling events leading to drought tolerance in vegetable-type soybean. 

## 4. Materials and Methods

### 4.1. Plant Material and Experimental Design

Five vegetable-type soybean (*Glycine max* L. Merrill) cultivars (UVE8, UVE17, UVE14, AGS354, AGS429) were planted in 9 L potting bags containing 10 kg of loamy sandy soil (one plant per pot). In a previous study [17], these cultivars were characterized according to their yield performance. AGS429 is a stable cultivar with low yield reduction under drought stress conditions; AGS354 and UVE8 are top-performing cultivars under optimal conditions but highly unstable under drought stress conditions; UVE14 is not a high yield performer but is stable under drought stress conditions; UVE17 is an unstable cultivar under drought stress conditions. A split-plot randomized complete block design trial with three replications was used. Each replication consisted of five pots. The trial was conducted under controlled environmental conditions (25 °C day and 18 °C night) in a greenhouse. Two water treatments, well-watered (at 100% soil water holding capacity) and severe stress (at 30% soil water holding capacity), were applied, according to the literature [28,29], at the third trifoliate stage. Pots were weighed daily for accurate water treatments. This was done in conjunction with the use of a Hydrosense II soil moisture sensor fitted with a CS659 (12 cm) portable soil-water probe (Campbell Scientific, Stellenbosch, South Africa) to ascertain the accuracy of the drought stress treatments. Young but fully expanded trifoliate leaves were harvested from each of the five pots per treatment, and this was replicated at flowering and pod filling stages. This was done because drought treatment at reproductive stages significantly increases the rate of pod abortion and, consequently, decreases seed yield [30]. The leaves were frozen in liquid nitrogen, combined (from each of the five pots per replication), and crushed in liquid nitrogen to form a homogenous fine powder, followed by storage at −26 °C. 

### 4.2. Proline Assay

A modified method of Carillo and Gobon [31] was used for the proline assay. Ethanol (4 mL) 70% (*v*/*v*) was used to homogenize the leaf powder (0.3 g) to a fine paste on ice. This step was repeated three times for each replication. The homogenate was centrifuged at 3000× *g* for 10 min. Supernatant (500 µL), 20% ethanol (500 µL), and 1% (*m*/*v*) acidic ninhydrin (prepared in 60%, *v*/*v*, glacial acetic acid) were vortexed before incubation at 95 °C for 20 min. After cooling, samples were centrifuged at 10,000× *g* for 10 min. The absorbance of the samples was measured at 520 nm (Cary 100 Bio, Varian, Australia) against a blank. A serial dilution of 0.016–0.16 mM proline (in 70% ethanol) was prepared and subjected to similar steps.

### 4.3. Total Soluble Sugars Assay

Determination of total soluble sugars (TSS) was done according to a method described by Irigoyen et al. [32]. Frozen leaf powder (0.1 g) was homogenized in 96% (*v*/*v*) ethanol, followed by incubation (80 °C, 10 min) and centrifugation (4000× *g*, 10 min, 4 °C). This procedure was repeated three times for each replication. Ethanoic extract (50 µL) was added to 1450 µL anthrone reagent (1.5 mg mL^−1^) prepared in 72% (*v*/*v*) sulphuric acid. The mixture was vortexed vigorously and incubated at 80 °C for 15 min. Absorbance was measured spectrophotometrically at 625 nm (Cary 100 Bio, Varian, Australia). The estimation of TSS was calculated from a glucose standard.

### 4.4. Lipid Peroxidation

Lipid peroxidation was done by measuring its by-product, malondialdehyde (MDA). The assay was done according to a modified method of Heath and Packer [33]. Leaf powder was homogenized in 5 mL 20% (*m*/*v*) trichloroacetic acid (TCA) and centrifuged (3500× *g*) for 20 min at 4 °C. This procedure was repeated three times for each replication. To 1 mL aliquot, 1 mL 20% (TCA) containing 0.5% thiobarbituric acid (TBA) was added. The mixture was vortexed, followed by incubation at 95 °C for 30 min. After cooling, the absorbance of the MDA-TBA product was measured spectrophotometrically at 532 and 600 nm (Cary 100 Bio, Varian, Australia). An extinction coefficient of 155 mM^−1^cm^−1^ was used to calculate the MDA content.

### 4.5. Hydrogen Peroxide Assay

A modified method described by Velikova et al. [34] was used for hydrogen peroxide (H_2_O_2_) content determination. To the frozen leaf powder (300 mg), 2 mL of ice-cold 0.1% trichloroacetic acid (TCA) (*w*/*v*) was added, homogenized, and centrifuged (12,000× *g*) for 15 min at 4 °C. This procedure was repeated three times for each replication. To the supernatant (0.5 mL), 0.5 mL 10 mM potassium phosphate buffer (pH 7.0) and 1 mL 1 M potassium iodide were added. For the blank, 0.1% TCA was used. Absorbance was measured at 390 nm (Cary 100 Bio, Varian, Australia). The H_2_O_2_ standard was subjected to similar conditions.

### 4.6. Enzyme Extract Preparation

Enzyme extracts were prepared in accordance with Pukacka and Ratajczak [35]. Leaf powder (0.5 g) for each treatment was homogenized to a fine paste on ice using a mortar and pestle in 5 mL 50 mM potassium phosphate buffer (pH 7.0) containing 1 mM EDTA, 2% PVPP, 0.1% Triton X-100, and 1 mM ascorbate. This procedure was repeated three times for each replication. The homogenate was centrifuged at 15,000× *g* for 20 min at 4 °C. The resulting aliquot was used as the enzyme extract. 

### 4.7. Enzyme Assays and Protein Content

The APX assay was performed according to a method described by Mishra et al. [36], with modifications. The reaction mixture (1 mL) consisted of 500 μL 50 mM phosphate buffer (pH 7.0), 200 μL 0.1 mM H_2_O_2_, 150 μL 0.5 mM sodium ascorbate, 50 μL 0.1 mM EDTA, and 100 μL enzyme. A decrease in absorbance as a result of ascorbate oxidation was measured at 290 nm (Cary 100 Bio, Varian, Australia) for 5 min at 20 °C against a blank in which the enzyme was replaced with phosphate buffer. An extinction coefficient of 2.8 mM^−1^cm^−1^ was used to calculate enzyme activity.

A modified method described by Zieslin and Ben-Zaken [37] was used for the determination of GPX. The reaction mixture contained 50 μL 0.2 M H_2_O_2_, 100 μL 50 mM guaiacol, 340 μL distilled H_2_O, 500 μL 80 mM phosphate buffer (pH 5.5), and 10 enzymes. An increase in absorbance as a result of tetraguaiacol formation was measured at 470 nm (Cary 100 Bio, Varian, Australia) for 3 min at 30 °C. The blank contained all reagents except for the enzyme, which was replaced with phosphate buffer. An extinction coefficient of 26.6 mM^−1^cm^−1^ was used to calculate enzyme activity. 

Protein concentration determination was done according to Bradford [38] using γ-globulin as a standard in order to calculate specific enzyme activities.

### 4.8. Yield Parameters

Yield traits (100 seed mass, total pods per plant, total seeds per plant, and total seed mass per plant) were measured at the end of the trial when the plants reached maturity (at the R8 growth stage). They were measured only to establish their relationships with the investigated biochemical responses, which better provide information for drought tolerance in vegetable-type soybean. The reason for not presenting them as part of this study is that a detailed study on how drought (severe and mild) affects these cultivars is already available [17].

### 4.9. Statistical Analysis

Data collected on all biochemical and yield parameters evaluated in this study were subjected to analysis of variance (ANOVA) in order to determine the separate and combined effects of cultivars and water treatments. A test for normality was done using the Shapiro–Wilk test. Where skewness in data was detected, data transformation was done using logarithmic (log10) transformation. Where significant effects in the ANOVA were detected, Fisher’s protected least significant difference (LSD) test at *p* = 0.05 was used to separate the means. Correlation analysis was performed to determine relationships between the biochemical and yield parameters. Principal component analysis (PCA) was performed to visualize associations between the parameters as well as genotype associations with the parameters. The PCA loadings biplot is a plot of the direction of vectors that define the model. They show how the original variables (the parameters in this study) contribute to creating the principal component (PC). The biplot shows the original parameters as vectors (colored lines). Orientation of the vector is indicated by the arrow, which further indicates the direction of the most variation and contribution to the respective PC. The angles between vectors of different parameters show their correlation in this space. For example, an acute angle between vectors represents a high positive correlation, a right angle represents a lack of correlation, and obtuse angles represent high negative correlations. All statistical analyses were performed using Genstat Release 19 software [39].

## 5. Conclusions

The levels of all biochemical responses (except H_2_O_2_) increased with the developmental stage in drought-stressed vegetable-type soybean. This study shows that total soluble sugars (TSS) at flowering and proline at pod filling are important to the drought tolerance responses of vegetable-type soybean. This prompts further investigations to identify specific non-structured sugars that are essential for drought tolerance improvements in this crop. Although the activities of the antioxidative enzymes increased under drought stress at both developmental stages in the drought-stable vegetable-type soybean cultivars (AG429 and UVE14), they are indirectly associated with drought tolerance because of their negative relationships with the yield parameters. This study suggests that they function in concert to minimize (but not completely eradicate) the excessive production of H_2_O_2_, thereby preventing excessive lipid peroxidation and membrane damage. A controlled increase in H_2_O_2_ production at both developmental stages under severe drought stress is crucial for the signaling events leading to an induction of the yield parameters, total seed mass per plant (TSMP) and total seed per plant (TSP), in vegetable-type soybean. A threshold for H_2_O_2_ to act as a signal molecule under drought stress in vegetable-type soybean needs thorough investigation because values between 3.09 and 4.14 mmol H_2_O_2_ g^−1^ fresh mass (in AGS354 and UVE8, respectively) promoted the highest lipid peroxidation, while lower values (2.65 mmol H_2_O_2_ g^−1^ fresh mass) seemed to stimulate signaling events. When thoroughly studied, such information could be useful for the application of low doses of peroxide for possible improvement of vegetable-type soybean production under severe drought stress. Although this study was performed in South Africa, it is applicable to the improvement of vegetable-type soybean drought tolerance globally.

## Figures and Tables

**Table 1:** Analysis of variance for the biochemical parameters of five vegetable-type soybean cultivars subjected to two water treatments at flowering and pod-filling stages.

		Flowering			Pod Filling	
Variate	Cultivar (C)	Treatment (T)	C × T	Cultivar (C)	Treatment (T)	C × T
APX	0.000697 ***	0.000669 ***	0.000429 ***	0.111300 ***	0.2875 ***	0.146200 ***
GPX	0.174360 ***	0.026590	0.102130 ***	0.114200 ***	0.0447 ***	0.009780 ***
GR	0.193300 ***	0.063000	0.127300 ***	0.331300 ***	0.000004	0.041500 *
H_2_O_2_	4.675600 ***	1.548600 **	0.075900	0.051778 ***	0.082577 ***	0.006332
MDA	0.068490 ***	0.053066 **	0.002256	15.70900 **	53.027 ***	2.737000
Proline	0.025750	0.006220	0.102680	0.191190 ***	0.1627 ***	0.017870
TSS	1.768700 ***	2.189700 ***	0.188200 *	0.256100	5.6472 ***	0.106800

* *p* ≤ 0.05, ** *p* ≤ 0.01, *** *p* ≤ 0.001. APX = ascorbate peroxidase, GPX = guaiacol peroxidase, GR = glutathione reductase, H_2_O_2_ = hydrogen peroxide, MDA = malondialdehyde, and TSS = total soluble sugars.

**Table 2:** Correlations between the biochemical and yield parameters for five vegetable-type soybean cultivars subjected to severe drought stress at flowering and pod-filling stages.

	Flowering				Pod Filling			
	100 SM	TPP	TSMP	TSP	100 SM	TPP	TSMP	TSP
APX	−0.2215	0.4857	−0.1685	−0.0100	0.2132	0.1394	−0.5640 *	−0.5636 *
GPX	−0.2366	0.4414	−0.2385	−0.0793	0.1640	0.2178	−0.0132	0.0906
GR	−0.3062	0.2359	−0.4766	−0.4130	0.0942	−0.0336	−0.6558 **	−0.6194 *
H_2_O_2_	−0.0122	0.4852	0.7846 **	0.6993 **	−0.0812	0.0417	0.4724	0.5615 *
MDA	0.3020	0.3745	0.7146	0.4747	−0.1244	0.0167	0.0489	0.1078
Proline	−0.3315	−0.2202	0.0749	0.2004	0.0111	0.2079	0.4955	0.6217 *
TSS	0.0541	0.515 *	0.4012	0.3894	0.2382	0.4506	0.4900	0.4846

* *p* ≤ 0.05, ** *p* ≤ 0.01. APX = ascorbate peroxidase, GPX = guaiacol peroxidase, GR = glutathione reductase, H_2_O_2_ = hydrogen peroxide, MDA = malondialdehyde, TSS = total soluble sugars, 100 SM = 100 seed mass, TPP = total pods per plant, TSMP = total seed mass per plant, and TSP = total seeds per plant.
